# Anti-inflammatory and cytoprotective effects of a squalene synthase inhibitor, TAK-475 active metabolite-I, in immune cells simulating mevalonate kinase deficiency (MKD)-like condition

**DOI:** 10.1186/s40064-016-3125-1

**Published:** 2016-08-30

**Authors:** Nobutaka Suzuki, Tatsuo Ito, Hisanori Matsui, Masayuki Takizawa

**Affiliations:** Pharmaceutical Research Division, Takeda Pharmaceutical Company Limited, 26-1 Muraoka-Higashi 2-chome, Fujisawa, Kanagawa 251-8555 Japan

**Keywords:** Squalene synthase inhibitor, MDIs, Mevalonate kinase deficiency patients, Anti-inflammatory effects, Cytoprotective effects

## Abstract

TAK-475 (lapaquistat acetate) and its active metabolite-I (TAK-475 M-I) inhibit squalene synthase, which catalyzes the conversion of farnesyl diphosphate (FPP) to squalene. FPP is a substrate for synthesis of other mevalonate-derived isoprenoids (MDIs) such as farnesol (FOH), geranlygeranyl diphosphate (GGPP), and geranylgeraniol. In patients with MKD, a rare autosomal recessive disorder, defective activity of mevalonate kinase leads to a shortage of MDIs. MDIs especially GGPP are required for prenylation of proteins, which is a posttranslation modification necessary for proper functioning of proteins like small guanosine triphosphatases. Malfunction of prenylation of proteins results in upregulation of the inflammatory cascade, leading to increased production of proinflammatory cytokines like interleukin-1β (IL-1β), eventually leading to episodic febrile attacks. In vitro, TAK-475 M-I incubation in a concentration dependent manner increased levels of FPP, GGPP, and FOH in human monocytic THP-1 cells. In subsequent experiments, THP-1 cells or human peripheral blood mononuclear cells (PBMCs) were incubated with simvastatin, which inhibits hydroxymethylglutaryl-coenzyme A reductase and thereby decreases levels of the precursors of MDIs, leading to the depletion of MDIs as expected in MKD patients. Increased levels of GGPP and FPP attenuated lipopolysaccharide (LPS)-induced IL-1β production in THP-1 cells and human PBMCs in statin-treated conditions. The MDIs also significantly reduced the damaged cell ratio in this active MKD-like condition. Moreover, TAK-475 M-I directly inhibited LPS-induced IL-1β production from statin-treated THP-1 cells. These results show anti-inflammatory and cytoprotective effects of MDIs via TAK-475 M-I treatment in statin-treated immune cells, suggesting that possible therapeutic effects of TAK-475 treatment in MKD patients.

## Background

Mevalonate kinase deficiency (MKD) is a rare periodic fever syndrome with autosomal recessive inheritance that is caused by a mutation in the *MVK* gene encoding mevalonate kinase (MK) (Esposito et al. 2014). MKD patients suffer from autoinflammatory disorders characterized by recurrent episodes of fever (Bader-Meunier et al. 2011) accompanied by painful lymphadenopathy; gastrointestinal symptoms, and so on (van der Hilst et al. 2008; van der Burgh et al. 2013; Ter Haar et al. 2013; Lainka et al. 2012). The *MK* gene mutation causes a dramatic decrease in MK enzymatic activity. MK converts mevalonic acid to mevalonate-5 phosphate, which is the precursor for the synthesis of a range of MDIs, including FPP, FOH, GGPP, GGOH, ubiquinones, and dicarboxylic acids (DCAs) (van der Burgh et al. 2013; Tricarico et al. 2014; Hubner et al. 1993; Xu et al. 2015). Consequently, MDI levels in MKD patients are reported to be decreased (Hubner et al. 1993). MDIs play a crucial role in the regulation and production of a range of cytokines, and decreased MDI levels are implicated in increases in pro-inflammatory mediators, including interleukin-1β (IL-1β), interleukin-6, C-reactive protein (CRP), and monocyte chemotactic protein-1 (Kostjukovits et al. 2015; Mandey et al. 2006; Marcuzzi et al. 2013). Moreover, an increased production of inflammatory cytokines by peripheral blood mononuclear cells (PBMCs), including monocytes, has been suggested as a central mechanism in the inflammatory phenotype of MKD.

**Scheme 1:**
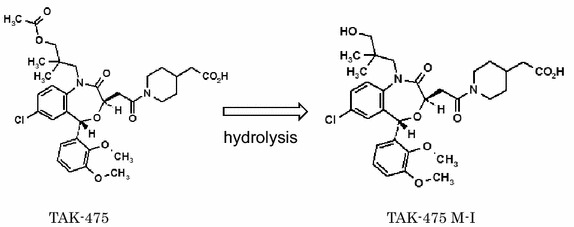
Chemical structures of TAK-475 and TAK-475 M-I. TAK-475, 1-{[(3*R*,5*S*)-1-(3-Acetoxy-2,2-dimethylpropyl)-7-chloro-5-(2,3-dimethoxyphenyl)-2-oxo-1,2,3,5-tetrahydro-4,1-benzoxazepin-3-yl]acetyl}piperidine-4-acetic acid (C_33_H_41_ClN_2_O_9_) is quickly hydrolyzed to TAK-475 M-I during the in vivo absorption process as shown in Scheme 1

**Scheme 2:**
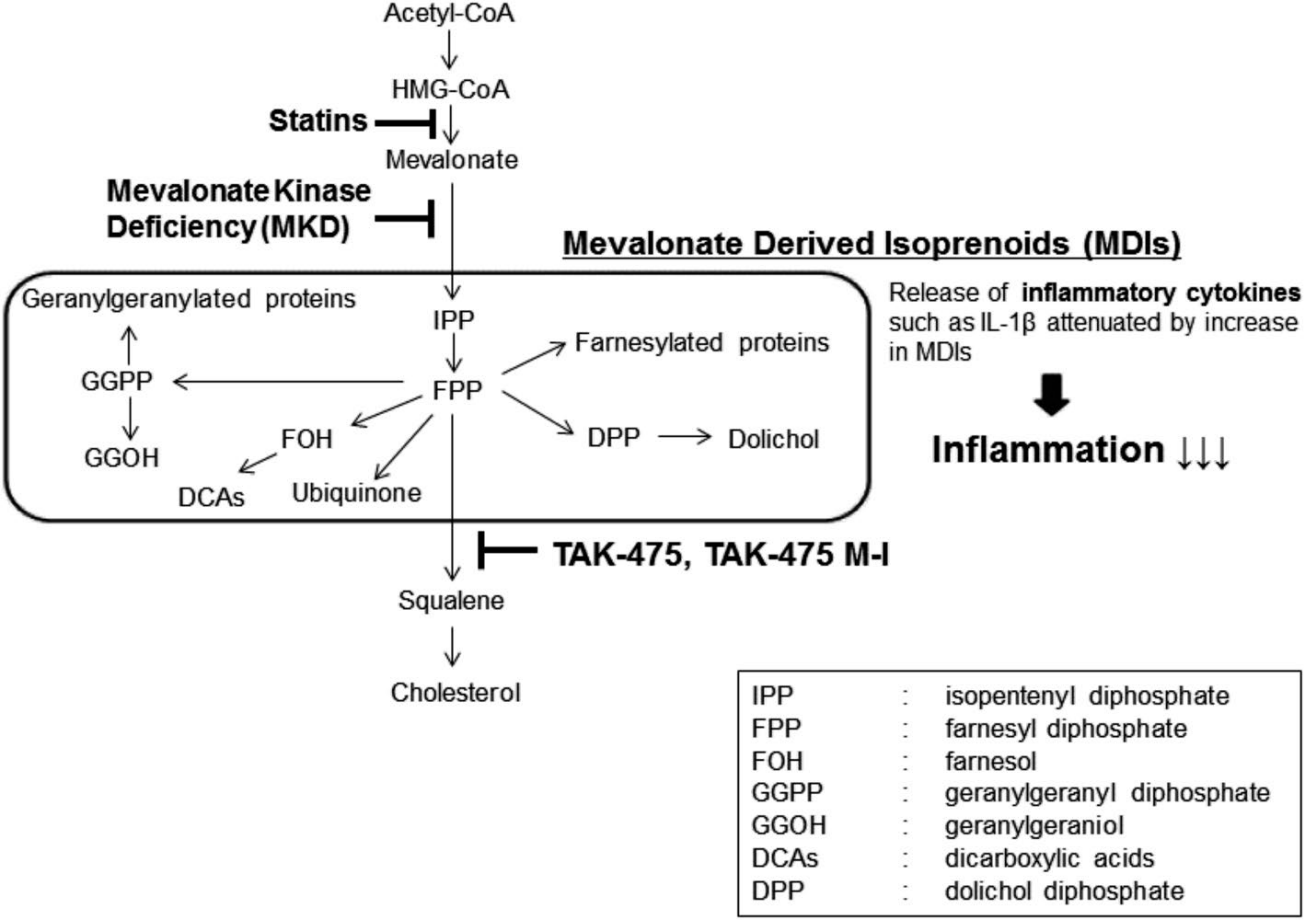
Mechanism of action of TAK-475 and TAK-475 M-I. Simply explaining, TAK-475 and TAK-475 M-I accumulate MDIs, which are strongly decreased in MKD patients, and the increased MDIs stop inflammation via blocking the release of inflammatory cytokines as shown in Scheme 2

TAK-475 (also termed as lapaquistat acetate) is a benzoxazepine derivative squalene synthase inhibitor (SSI) (Miki et al. 2002; Nishimoto et al. 2003), which was developed by Takeda Pharmaceutical Company Limited and has proceeded to clinical Phase III in hyperlipidemia indication (Stein et al. 2011). In this Phase III clinical trial, the dose-dependent cholesterol-lowering efficacy via inhibition of squalene synthase by TAK-475 was indeed observed with occasional liver enzyme elevation, ultimately leading to the discontinuation of clinical development in 2007 based on the risk–benefit evaluation. Preclinical pharmacokinetic studies have demonstrated that most of the dosed TAK-475 is quickly hydrolyzed to TAK-475 M-I during the in vivo absorption process (Miki et al. 2002) as shown in Scheme 1. We hypothesized the inhibition of squalene synthase by TAK-475 could increase the level of MDIs in MKD patients (Scheme 2). Increased MDIs would in turn attenuate the unregulated inflammatory cascade and eventually lead to the overall improvement of their clinical symptoms. In support of our hypothesis, it has been reported that another squalene synthase inhibitor, zaragozic acid A, on skin fibroblasts from MK-deficient patients has shown increased residual MK enzyme activity, indicating that treatment of MKD with the supply of MDIs may prove beneficial in the prevention and treatment of the symptoms of MKD (Schneiders et al. 2006). In this report we have investigated the effects of TAK-475 M-I on the levels of MDIs as well as anti-inflammatory properties in MKD-like immune cells in order to evaluate whether TAK-475 could confer clinical benefit in patients with MKD.

## Methods

### Chemicals

TAK-475 M-I (T-0091485, Lot No. X000000090965) was synthesized in Chemical Development Laboratories of Takeda Pharmaceutical Company Limited. For internal control, GGPP (Lot No. E00166-66) and FPP (Lot No. MJB-E00026-28) were purchased from Echelon Biosciences. GGOH (Lot No. 2598758) was purchased from LKT Laboratories. FOH (Lot No. MKBK0393 V) was purchased from Sigma-Aldrich. LPS (Lot No. PDM3922) were purchased from Wako Pure Chemical Industries.

### Cell cultures and treatments

**Fig. 1:**
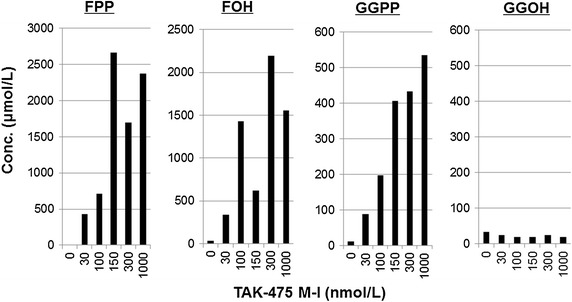
FPP, FOH and GGPP but not GGOH were increased in a dose-dependent trend in 48 h treatment of TAK-475 M-I. THP-1 cells were stimulated with various concentrations of TAK-475 M-I for 48 h. FOH, FPP, GGOH and GGPP in the cells were quantified by LC–MS/MS

**Fig. 2:**
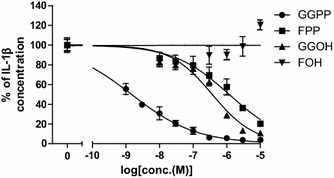
GGPP, FPP and GGOH inhibited LPS-stimulated IL-1β production in MKD-like THP-1 cells. IL-1β protein concentration in culture supernatants of LPS-stimulated MKD-like THP-1 monocyte cells was measured using ELISA. Data are represented as mean ± SD, n = 3

**Fig. 3:**
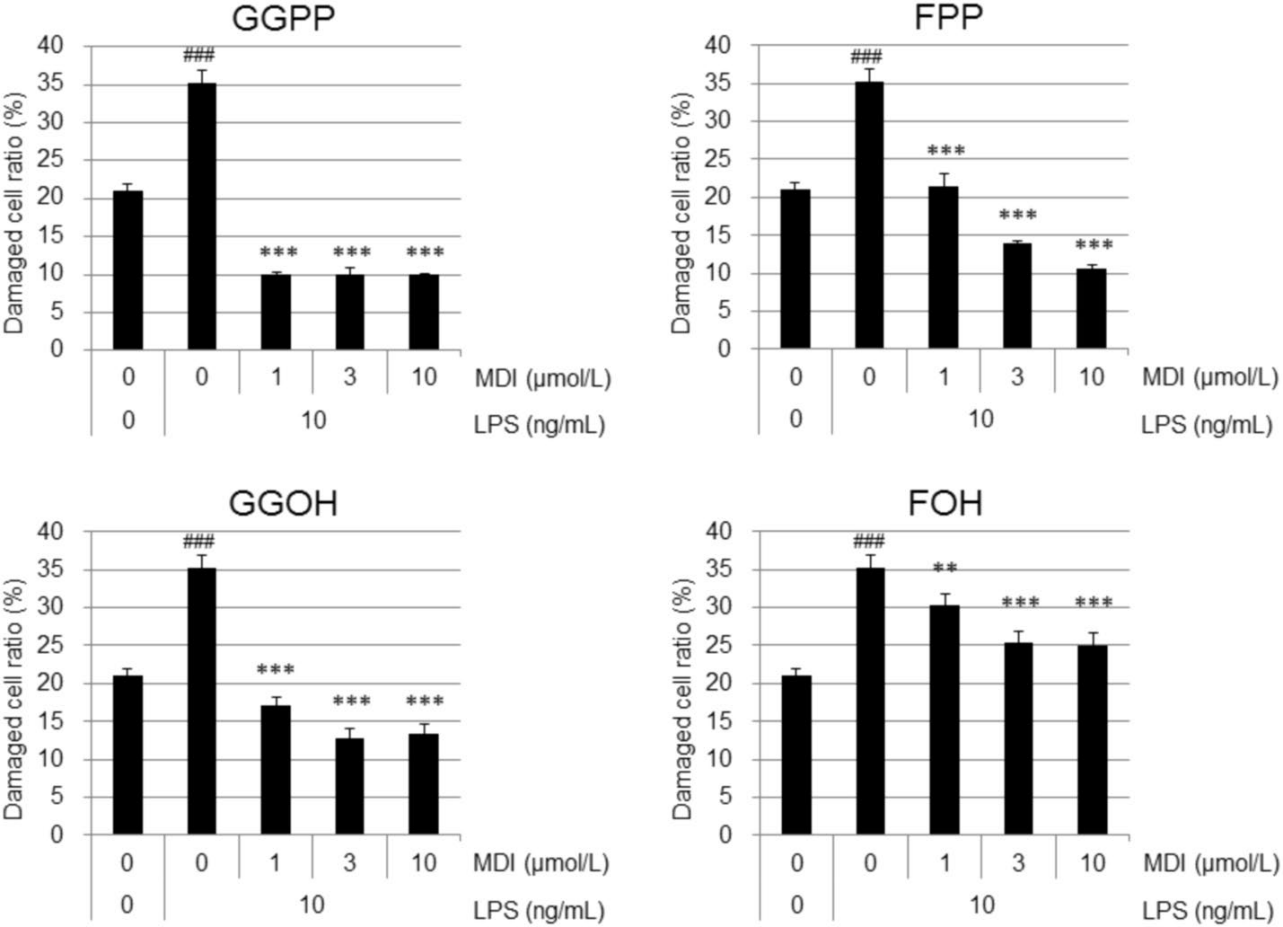
MDIs significantly inhibited cytotoxicity measured by DCR in LPS-stimulated MKD-like THP-1 cells. Damaged cell ratio was measured in triplicates using ToxiLight bioassay kit (Lonza, Basel, Switerland) and expressed the ratio of AK activity from AK-releasing cells against that from total cells. Data are represented as mean + SD, n = 3. The cytotoxicity of LPS was evaluated by a Student’s t-test with significance set at ^###^ *P* < 0.001 (versus 0 μmol/L of MDI and 0 ng/mL of LPS). The dose-dependent effects of MDIs in LPS-stimulated MKD-like THP-1 cells were evaluated by a two-tailed Williams’ test with significance set at **P < 0.01 and ***P < 0.001 (versus 0 μmol/L of MDI and 10 ng/mL of LPS)

THP-1 human monocytic cells (ATCC No. TIB-202) were obtained from American Type Culture Collection (ATCC, Manassas, VA, USA). THP-1 cells were grown in RPMI 1640 supplemented with 10 % FBS (Life Technologies) and penicillin–streptomycin (Wako Pure Chemical Industries) at 37 °C, 5 % CO_2_ in humidified incubator(Liao et al. 2013). THP-1 cells were stimulated with various concentrations of TAK-475 M-I for 48 h (Fig. 1). Or THP-1 cells were treated with 4 μmol/L of simvastatin for 24 h, following stimulated with 10 ng/mL of LPS in the presence of MDIs at concentrations from 0.001 to 10 μmol/L for additional 24 h (Figs. 2, 3). Or THP-1 cells were treated with simvastatin (3 or 4 μmol/L) for 48 h in the presence of TAK-475 M-I (150 or 1000 nmol/L), following stimulated with LPS (0.1, 1 or 10 ng/mL) for additional 24 h (Fig. 6). Then, supernatants or cells pellets were collected and stored at −80 °C before the measurement of the concentrations of IL-1β or MDIs.

### Human PBMCs isolation and treatment

**Fig. 4:**
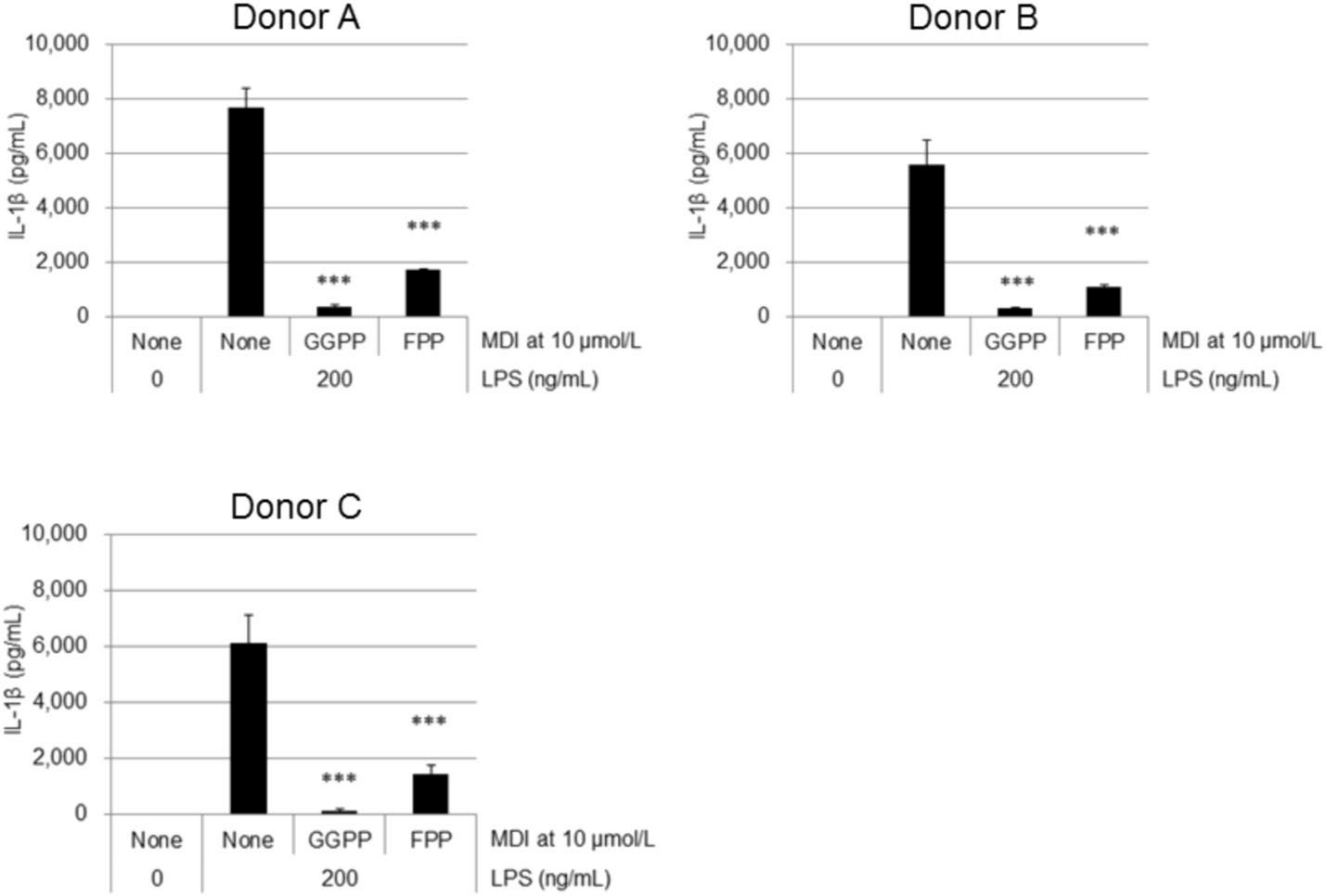
GGPP and FPP inhibited LPS-stimulated IL-1β production in MKD-like human PBMCs. IL-1β protein concentration in culture supernatants of LPS-stimulated MKD-like PBMCs in the presence of MDIs (both: 10 μmol/L) was measured in triplicates using ELISA. Data are represented as mean + SD, n = 3. Statistical significance was determined by a Dunnett’s test. ***P < 0.001 versus 0 μmol/L of MDI with LPS

**Fig. 5:**
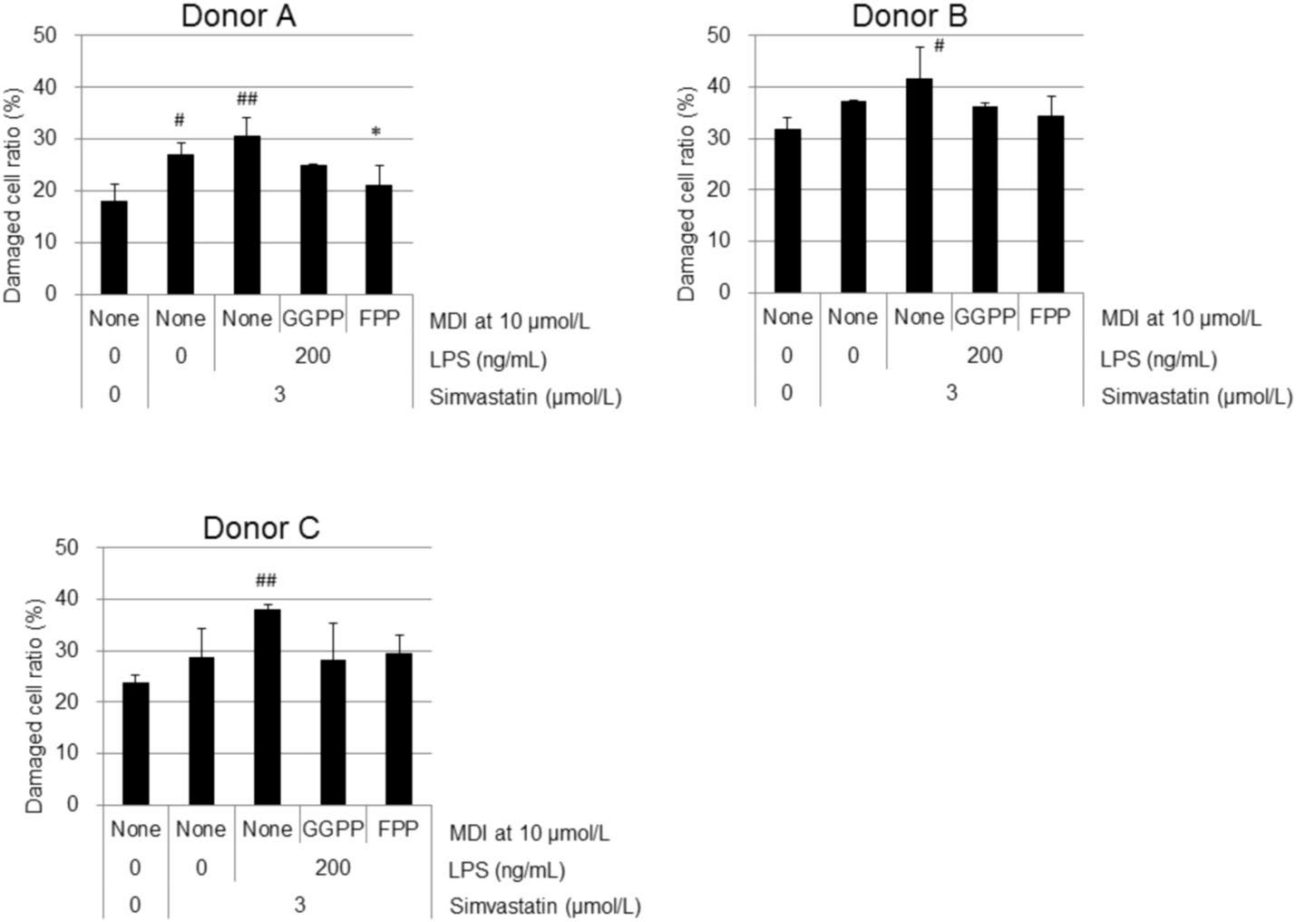
GGPP and FPP showed a tendency to have cytoprotective effects in LPS-stimulated MKD-like human PBMCs. Damaged cell ratio (DCR) was measured using ToxiLight bioassay kit (Lonza) and expressed the ratio of AK activity from AK-released cells against that from total cells. Data are represented as mean + SD, n = 3. The cytotoxicity of simvastatin and LPS in PBMCs was evaluated by a Dunnett’s test with significance set at ^#^ *P* < 0.05 and ^##^ *P* < 0.01 (versus 0 μmol/L of MDI without simvastatin and LPS). The effects of GGPP and FPP in LPS-stimulated MKD-like PBMCs were evaluated by a Dunnett’s test. *P < 0.05 (versus 0 μmol/L of MDI with LPS and simvastatin)

Human whole blood was collected from 3 healthy volunteer donors. All human healthy volunteer donors have provided their informed consent for participation, in compliance with all Principles of the Declaration of Helsinki. This study was approved by Independent Ethics Committee of Takeda Pharmaceutical Company Limited, Kanagawa, Japan. According to the standard Ficoll-Paque density gradient centrifugation methods, human PBMCs were collected and grown in 96 well plates (2 × 10^5^/well) with culture medium at 37 °C, 5 % CO_2_ in humidified incubator. Human PBMCs were treated with 3 μmol/L of simvastatin for 24 h. Then, the cells were stimulated with 200 ng/mL of LPS in the presence of 10 μmol/L of GGPP or FPP for additional 24 h (Figs. 4, 5).

### Measurements of the concentration of MDIs

FOH, FPP, GGOH and GGPP in cells were quantified by LC–MS/MS by Sumika Chemical Analysis Service., and the lowest detection limit was 0.008 μg/1.5 × 10^6^ cells, and the highest detection limit was 2 μg/1.5 × 10^6^ cells. Values below the detection limit were presumed to be 0.008 μg/1.5 × 10^6^ cells, and values higher the detection limit were presumed to be 2 μg/1.5 × 10^6^ cells.

### Extraction and enzymatic hydrolysis

In cell samples (1.5 × 10^6^ cells), 100 μL of 8 % KOH ethanol solution was added and the samples were incubated at 80 °C for 2 h. The samples were agitated during the incubation to disintegrate the plasma and cell efficiently. The obtained solution was cooled down to ambient temperature and centrifuged at 3000 rpm for 5 min. To the obtained supernatant (50 μL), 10 μL of *n*-pentadecanol (I.S.) solution (1 μg/mL) and 2 mL of 1.5 mol/L Tris–HCl buffer (pH8.6) containing 0.25 mol/L MgCl_2_ were added. FOH, GGOH and I.S. in the solution were extracted with 5 mL of *n*-hexane/ethanol (98.5:1.5, v/v, 2 times), and the obtained supernatant by centrifugation at 3000 rpm for 5 min was evaporated under a stream of N_2_ gas. FPP and GGPP were quantified as FOH and GGOH, respectively (C_total_) together with free FOH and GGOH (C_free_) after enzymatic hydrolysis using alkaline phosphatase. The concentrations of FPP and GGPP were calculated by subtracting the C_free_ from C_total_. If the calculated value is negative, the concentration is described as “0.00”. For enzymatic hydrolysis, alkaline phosphatase (1 unit) were added to 8 % KOH sample solution (plasma: 20 μL, cell: 50 μL) with 2 mL of 1.5 mol/L Tris–HCl buffer (pH 8.6) containing 0.25 mol/L MgCl_2_, and samples were incubated at 37 °C for 2 h. The following extraction procedure was the same as described above.

### Derivatization of FOH and GGOH

To above extracts, 250 μL of 3-nitrophthalic anhydride pyridine solution (10 mg/mL) was added, and the samples were incubated at 70 °C for 30 min. After the incubation, pyridine was evaporated under a stream of N_2_ gas. The residue was dissolved in 200 μL of methanol, and 500 μL of 50 mmol/L phosphate buffer (pH3) was added to the methanol solution. The derivatives of FOH, GGOH and I.S. (FOH-NPA, GGOH-NPA and I.S.-NPA respectively) in the solution were extracted with 5 mL of *n*-hexane. *n*-hexane extract was concentrated under the N_2_ gas, the residue was dissolved in a mixture of methanol/water/acetic acid (75:25:0.1, v/v/v) (200 μL) for LC–MS/MS analysis. A 10 μL portion of the solution was injected into the LC–MS/MS system.

### LC–MS/MS

FOH-NPA, GGOH-NPA, and I.S.-NPA were analyzed using a LC-10A system (Shimadzu Corp., Kyoto, Japan) coupled with API4000 (AB Sciex Pte. Ltd., Ontario, Canada). The column was used Symmetry Shield RP8 (2.1 mm ID × 150 mm L., Waters, MA, USA). A mixture of acetonitrile/water/acetic acid (90:10:0.1, v/v/v) was used as a mobile phase at a flow rate of 0.2 mL/min. The conditions of turbo ion spray interface and mass spectrometer were operated under the following conditions: ionization polarity, negative; ion spray voltage, −4.5 kV; turbo probe temperature, 425 °C; curtain gas flow, 30 L/min (N_2_); nebulizer gas flow, 40 L/min (air); heater gas flow (N2), 70 L/min; and collision gas pressure (N2), 10 mPa. Spectra were obtained in the selected reaction monitoring (SRM) mode. The monitoring ions and collision energy were shown as follows:

$${\text{FOH-NPA}}\quad m/z414\;to\;m/z\;166 \quad- 20\;{\text{eV}}$$

$${\text{GGOH-NPA}}\quad m/z\;482\;to\;m/z\;166 \quad- 22\;{\text{eV}}$$

$${\text{I}}.{\text{S}}.{\text{-NPA}}\quad m/z\;420\; \, to\;m/z\;166 \quad- 22\;{\text{eV}}$$

Peaks on the chromatogram were identified based on the retention time and the mass-to-charge ratio (*m/z*) of the monitoring ions. The concentrations of FOH and GGOH were determined from the peak area of FOH and GGOH using the absolute calibration curve method because I.S. peak area was varied widely. The calibration curves of FOH and GGOH were linear in the spiked concentration range from 0.008 to 0.8 μg/mL.

### Measurements of IL-1β protein concentration by ELISA and statistics

**Fig. 6:**
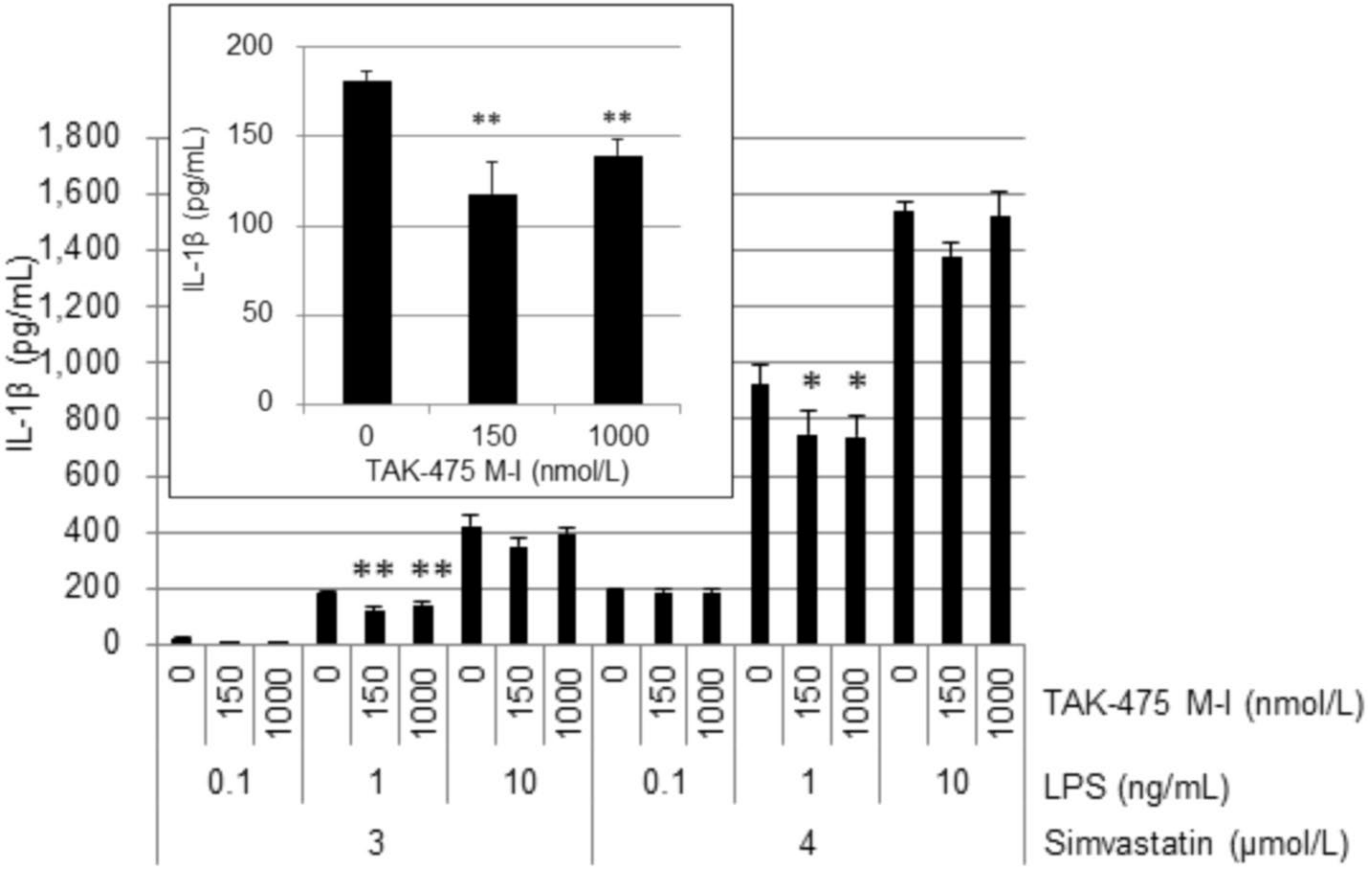
TAK-475 M-I inhibited LPS-stimulated IL-1β production in MKD-like THP-1 cells. IL-1β protein concentration in culture supernatants of LPS-stimulated MKD-like THP-1 cells was measured using ELISA. Inserted figure is an enlarged view of the region at 3 μmol/L simvastatin and 1 ng/mL of LPS. Data are represented as mean + SD, n = 3. Statistical significance was determined by a Williams’ test with significance set at *P < 0.025 and **P < 0.005 (versus 0 nmol/L of TAK475 M-I in each experimental condition). ND: not detected

The concentration of IL-1β protein in culture supernatants was measured in triplicates using ELISA kits (R&D Systems, Minneapolis, MN, USA), according to the manufacturer’s instruction. The lower limit of quantification (LLOQ) was 7.8 pg/mL, and measurement values below the LLOQ were presumed to be 7.8 pg/mL. Data are presented as the mean ± SD. IC50 values were calculated by GraphPad Prism ver.5 from the data expressed as % of IL-1β protein concentration. The mean IL-1β concentration of vehicle control without LPS was set as the 0 %, and the mean IL-1β concentration of vehicle control with LPS was set as the 100 %. For the analysis of inhibitory effects of GGPP and FPP on LPS-stimulated IL-1β production, statistical significance was determined by a Dunnett’s test. *P* < 0.05 was considered statistically significant (Fig. 4). For dose–response analysis of inhibitory effect of TAK-475 M-I on LPS-stimulated IL-1β production, statistical significance was determined by a one-tailed Williams’ test. *P* < 0.025 was considered statistically significant (Fig. 6).

### Cytotoxicity assay and statistics

Cytotoxicity assay was conducted using ToxiLight bioassay kit (Lonza, Basel, Switzerland), a bioluminescent, non-destructive cytolysis assay kit designed to measure the release of the enzyme, adenylate kinase (AK), from damaged cells. Damaged cell ratio (DCR) was estimated by dividing AK activity from AK-released cells by that from total cells. Data are represented as the mean + SD (n = 3). The cytotoxicity of LPS was evaluated by a Student’s *t* test, and the dose-dependent effects of MDIs in LPS-stimulated MKD-like THP-1 cells were evaluated by a two-tailed Williams’ test. In both tests, *P* < 0.05 was considered statistically significant (Fig. 3). The cytotoxicity of simvastatin and LPS in THP-1 cells or the effects of GGPP and FPP in LPS-stimulated MKD-like THP-1 cells were evaluated by a Dunnett’s test. *P* < 0.05 was considered statistically significant (Fig. 5).

## Results

### TAK-475 M-I significantly increased the levels of MDIs in THP-1 cells

Effects of TAK-475 M-I on the level of four MDIs; FPP, FOH, GGPP and GGOH in human monocytic THP-1 cells were examined. THP-1 cells were treated for 48 h with 0, 30, 100, 150, 300 and 1000 nmol/L of TAK-475 M-I. The concentrations of MDIs were calculated assuming that 10^6^ THP-1 cell volume is equal to 970 μL (Kuijk et al. 2008). Levels of FPP and GGPP were increased by 48 h in a dose-dependent manner, whereas GGOH levels did not show any apparent changes (Fig. 1). Although there is a poor dose-dependency in FOH induction, a trend is observed in that high concentration of TAK-475 M-I can induce more FOH (Fig. 1). TAK-475 M-I potently inhibited de novo cholesterol synthesis in human primary hepatocytes with relative IC_50_ values of 110 nmol/L (unpublished data). It should be obviously difficult to detect cholesterol level in human monocytic THP-1 cells compared with human hepatocytes because cholesterol is mainly synthesized in the liver. If TAK-475 M-I can inhibit cholesterol synthesis in THP-1 cells like in human primary hepatocytes, TAK-475 M-I is suggested to be able to strongly increase FPP, FOH and GGPP with the effect against cholesterol inhibition.

### MDIs blocked LPS-stimulated IL-1β production in MKD-like THP-1 cells

Effects of MDIs on LPS-stimulated IL-1β production in MKD-like human monocytic THP-1 cells were investigated. THP-1 cells were pretreated with simvastatin (4 μmol/L) for 24 h in order to induce MKD-like phenotype by reducing endogenous MDI levels (Galeotti et al. 2012). The pretreated THP-1 cells were then treated with various concentrations of GGPP, GGOH, FPP, or FOH in the presence of LPS (10 ng/mL) for an additional 24 h. GGPP, GGOH and FPP showed dose-dependent suppression in IL-1β production in the culture supernatants (Fig. 2), and half maximum inhibitory concentration (IC_50_) values were 1.5, 369 and 1179 nmol/L, respectively (Table 1). Among these MDIs, GGPP had the most potent inhibitory effect. FOH did not show significant changes in IL-1β release. This study demonstrated that exogenous supply of GGPP, FPP and GGOH can strongly suppress LPS-induced IL-1β production in MKD-like THP-1 cells.

**Table 1:** The mean IC_50_ of MDIs for the inhibition of LPS-stimulated IL-1β production in MKD-like THP-1 cells

**MDI**	IC50 (nmol/L)	95 % confidence interval
GGPP	1.5	1.1–1.9
FPP	1179	856–1625
GGOH	369	282–483
FOH	ND	ND

*ND* not determined

### MDIs also reduced cytotoxicity in LPS-stimulated MKD-like THP-1 cells

Effects of MDIs on cytotoxicity in LPS-stimulated MKD-like human monocytic THP-1 cells were investigated. The treatment of 10 ng/mL of LPS increased DCR from approximately 20–35 % (Fig. 3). MDIs showed inhibitory effects against LPS-induced cytotoxicity in statin-treated THP-1 cells (Fig. 3). GGPP reduced LPS-induced DCR by approximately 70 % even at 1 μmol/L concentration, because 35 % DCR became 10 % DCR by shaving off 25 % worth of cytotoxicity which is about 70 % of LPS-induced DCR. FPP also reduced LPS-induced DCR by approximately 70 % at 10 μmol/L concentration. Moreover, other MDIs such as GGOH and FOH can also significantly reduce LPS-induced cytotoxicity in MKD-like condition (Fig. 3).

These results has shown in Figs. 2 and 3 suggest that external supply of GGPP, FPP and GGOH suppresses the production of IL-1β without cytotoxicity.

### MDIs blocked LPS-stimulated IL-1β production in MKD-like human PBMCs

Effects of GGPP and FPP on LPS-stimulated IL-1β production in statin-treated human PBMCs were investigated. Human PBMCs were pretreated with simvastatin (3 μmol/L) for 24 h to induce an MKD-like phenotype by reducing endogenous MDI levels (Kuijk et al. 2008), and then stimulated with LPS (200 ng/mL) in the presence of GGPP or FPP (10 μmol/L) for an additional 24 h. Both GGPP and FPP at 10 μmol/L significantly inhibited LPS-stimulated IL-1β production in MKD-like human PBMCs in all 3 donors (Fig. 4). It was obvious that GGPP had a much stronger inhibitory effect compared with FPP, confirming the IC_50_ value on Table 1 (Fig. 4).

### MDIs also showed a tendency to reduce cytotoxicity in LPS-stimulated MKD-like human PBMCs

DCR of PBMCs increased with the treatment of simvastatin and LPS (Fig. 5). However, GGPP or FPP (10 μmol/L) showed a tendency to decrease the DCR in all donors. Judging from Figs. 4 and 5, both GGPP and FPP at 10 μmol/L significantly inhibited LPS-stimulated IL-1β production without cytotoxicity in MKD-like human PBMCs.

### TAK-475 M-I suppresses LPS-stimulated IL-1β production in both MKD-like THP-1 cells and human PBMCs

**Fig. 7:**
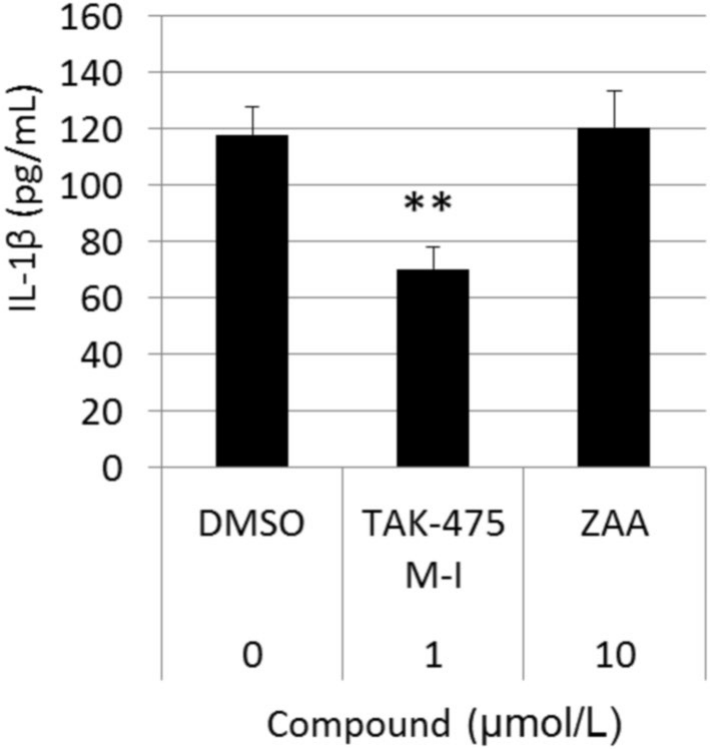
TAK-475 M-I inhibited LPS-stimulated IL-1β production in MKD-like human PBMCs. IL-1β protein concentration in culture supernatants of LPS-stimulated MKD-like human PBMCs was measured using ELISA. The experiment was conducted at 3 μmol/L simvastatin and 8 ng/mL of LPS. Data are represented as mean + SD, n = 3. Statistical significance was determined by a Dunnetts’ test with significance set at **P < 0.01 (vs. Control). ZAA: zaragozic acid A

Effects of TAK-475 M-I on LPS-stimulated IL-1β production in statin-treated human monocytic THP-1 cells were investigated. THP-1 cells were treated with simvastatin (3 or 4 μmol/L) to induce MKD-like phenotype by reducing endogenous MDI levels (Kuijk et al. 2008) and with TAK-475 M-I (150 or 1000 nmol/L) for 48 h, and then stimulated by LPS (0.1, 1 or 10 ng/mL) for additional 24 h. Both 150 and 1000 nmol/L of TAK-475 M-I significantly reduced LPS-stimulated IL-1β production in MKD-like THP-1 cells under the condition of 1 ng/mL LPS plus 3 μmol/L simvastatin (top-left balloon of Fig. 6). Similar trends were observed in other experimental conditions, but the effects were not always statistically significant under the condition of 0.1 or 10 ng/mL LPS (Fig. 6). Inhibitory effects of TAK-475 M-I at a concentration of 150 and 1000 nmol/L appeared to be comparable under the condition of 1 ng/mL LPS plus 3 or 4 μmol/L simvastatin (Fig. 6). This suggests that it reached a plateau even in 150 nmol/L. TAK-475 M-I did not show significant cytotoxicity in LPS-stimulated MKD-like THP-1 cells at a concentration of 1000 nmol/L (data not shown). Moreover, 1000 nmol/L of TAK-475 M-I reduced 41 % of LPS-stimulated IL-1β production even in MKD-like human PBMCs under the condition of 8 ng/mL LPS plus 3 μmol/L simvastatin (Fig. 7). These results suggest that TAK-475 M-I also suppresses the production of IL-1β, which is one of causes of fever episode of MKD patients (Galeotti et al. 2012) without cytotoxicity.

## Discussion

TAK-475 and its active metabolite M-I inhibit the enzyme that catalyzes the conversion of FPP to squalene on the cholesterol biosynthesis pathway. TAK-475 has been evaluated extensively in both in vitro and in vivo experimental models that have confirmed cholesterol-lowering properties (Nishimoto et al. 2003; Amano et al. 2003). In patients with MKD, defective activity of the MK enzyme is hypothesized to lead to a shortage of MDIs such as FPP. Through squalene synthase inhibition in the cholesterol biosynthesis pathway, TAK-475 disrupts the conversion of FPP into squalene resulting in increased levels of FPP and other MDIs.

The effects of TAK-475 M-I on MDIs were examined in in vitro experiments. These results showed that treatment with TAK-475 M-I resulted in the significant increase of MDIs such as FPP, FOH, and GGPP in human monocytic THP-1 cell line, as shown in Fig. 1. In subsequent studies potential inhibitory effects of MDIs on IL-1β secretion were investigated in MKD-like conditions. THP-1 cells and PBMCs were first incubated for 24 h with simvastatin, which inhibits hydroxymethylglutaryl-coenzyme A (HMG-CoA) and thereby decreases levels of the precursors for MDI production (Bellosta et al. 2000), leading to MDI depletion as expected in MKD patients. The cells were then stimulated for 24 h with LPS, which induces IL-1β secretion, while in the presence of MDIs. Results from this study demonstrated that pretreatment of FPP, GGPP, and GGOH in a concentration-dependent manner decreased LPS- induced IL-1β secretion in MKD-like THP-1 cell lines (Fig. 2). Similarly, GGPP and FPP both decreased LPS-induced IL-1β secretion in MKD-like human PBMCs regardless of the donor’s source (Fig. 4). The production of inflammatory cytokines such as IL-1β is considered as one of the causes of fever episodes in MKD patients (Galeotti et al. 2012). The increased levels of MDIs after treatment with TAK-475 are therefore expected to mitigate the severity and/or frequency of fever episodes in patients with MKD.

According to the human plasma PK profile of TAK-475, oral administration of TAK-475 100 mg once daily leads to the average plasma concentration of approximately 30 nmol/L of total active concentration of TAK-475 and its active metabolites (data not shown). Therefore, based on the detailed data in Fig. 1, it is anticipated that 30 nmol/L of TAK-475 can induce approximately 427 µmol/L of FPP and approximately 88 µmol/L of GGPP in THP-1 cells. The anticipated increase in levels of MDIs may be much less in MKD patients compared to healthy human subjects because of very limited availability of isopentenyl pyrophosphate (IPP). However, given that the mean inhibitory concentrations (IC_50_) of FPP and GGPP are very low at 1.18 µmol/L and 1.5 nmol/L, respectively, in MKD-like THP-1 cells (Table 1), a slight increase in FPP and GGPP levels induced by TAK-475 100 mg may give sufficient inhibition of IL-1β production in MKD patients and mitigate fever episodes. Moreover, treatment with TAK-475 M-I was confirmed to inhibit IL-1β production in THP-1 cell lines exhibiting an MKD-like phenotype in 35 % reduction at 150 nmol/L and 23 % reduction at 1000 nmol/L (Fig. 6). It is also confirmed that TAK-475 M-I can inhibit IL-1β production even in MKD-like human PBMCs in 41 % reduction at 1000 nmol/L (Fig. 7). Increased MDIs by TAK-475 treatment would be able to block the production of inflammatory cytokines, which is one of the causes of fever episodes in MKD patients, in the clinical setting (Marcuzzi et al. 2013; Galeotti et al. 2012). Similarly, Kuijk et al. (2008) showed that the addition of GGPP led to a decrease in levels of IL-1β in THP-1 cell lines that were treated with simvastatin to produce MKD-like inflammatory phenotype.

Interestingly, MDIs have significant cytoprotective effects in LPS-stimulated THP-1 cells, which are treated with simvastatin to induce an MKD-like phenotype. It is reported that statins induce apoptosis via caspase-3 activation in glioma cells (Yanae et al. 2011). As LPS stimulation is also known to induce apoptosis in some endothelial cells, it would be reasonable that LPS treatment enhanced DCR 75 % more in MKD-like THP-1 cells by changing from 20 % DCR into 35 % DCR (Fig. 3). This DCR was most significantly inhibited by GGPP as shown Fig. 3. This might be due to the inhibition caspase-3 activity via ERK1/2 and Akt activations because the supplementation of MDIs such as GGPP is reported to enhance both Ras-ERK1/2 and Ras-Akt signaling pathways (Yanae et al. 2011). Similarly, the supplementation of both GGPP and FPP decreased LPS-induced DCR in statin-treated human PBMCs (Fig. 5). These results suggested that TAK-475 could recover the damaged cells of MKD patients, which were caused by the decreases levels of the precursors for MDI production.

Our results suggest that TAK-475 M-I can block the production of inflammatory cytokine IL-1β as well as significantly enhanced the cytoprotective effect in MKD-like human immune cells, indicating that TAK-475 has a potential to be the disease modifying drug in patients with MKD.

## Conclusion

In this study, we have demonstrated the anti-inflammatory and cytoprotective potentials of TAK-475 M-I, benzoxazepine derivative SSI, through the accumulation of MDIs in immune cells simulating MKD-like condition. As the shortage of MDIs could be the main cause of MKD symptoms such as fever episodes, the disease-modifying therapeutic effects will be expected by TAK-475 treatment in MKD patients.
